# Cervical cancer screening behavior and associated factors among women of Ugrachandi Nala, Kavre, Nepal

**DOI:** 10.1186/s40001-017-0274-9

**Published:** 2017-09-19

**Authors:** Radha Acharya Pandey, Era Karmacharya

**Affiliations:** Kathmandu University School of Medical Sciences, Dhulikhel Hospital, Kathmandu University Hospital, Dhulikhel, Kavre Nepal

**Keywords:** Cervical cancer, Screening, Health belief model scale

## Abstract

**Background:**

Cervical cancer in Nepal ranks as the first most frequent cancer among women. Primary prevention measures, such as prophylactic vaccines against high risk HPV, are now available. Over time, vaccination will decrease the prevalence of the disease among younger women; however, screening will still be needed. The objective of the study was to assess the cervical cancer screening behavior and its associated factors among women of Nala Village Development Committee (VDC), Kavre.

**Methods:**

A descriptive cross-sectional study was done to assess the cervical cancer screening behavior among women in 2014. Systematic Random sampling was used to collect the data from a sample of 180 women residing in Nala VDC. A structured interview questionnaire and health belief model scale was used to collect data. Descriptive and inferential statistics (Chi-square test) was used for data analysis using SPSS version 16 program.

**Results:**

Minority (18.3%) of the respondents had cervical cancer screening behavior. Education level of the respondents was significantly associated with cervical cancer screening behavior (*p* < 0.05). Age, parity, perceived susceptibility, perceived benefits, and perceived barriers had no significant association with cervical cancer screening behavior.

**Conclusion:**

This study shows that cervical cancer screening behavior was satisfactory. The findings of the study indicate a significant association between cervical cancer screening behavior and education level of the participants. Awareness campaigns targeting illiterate groups can be conducted in community so that they become motivated towards cervical cancer screening.

## Background

With 528,000 new cases every year, cervical cancer is the fourth most common cancer affecting women worldwide, after breast, colorectal, and lung cancers. A large majority (around 85%) of the global burden occurs in the less developed regions, where it accounts for almost 12% of all female cancers [1].

It is also the fourth most common cause of cancer death (266,000 deaths in 2012) in women worldwide accounting for 7.5% of all female cancer deaths. Almost nine out of ten (87%) cervical cancer deaths occur in the less developed regions [1].

According to GLOBOCAN, there were an estimated 175,000 new cases of cervical cancer and 94,000 deaths in South-East Asia Region with 5-year prevalence of 465,000 cases of cervical cancer in 2012 [1].

Nepal has a population of 9.65 million women aged 15 years and older who are at risk of developing cervical cancer. Current estimates indicate that every year 2332 women are diagnosed with cervical cancer and 1367 die from the disease. Cervical cancer in Nepal ranks as the first most frequent cancer among women and the first most frequent cancer among women between 15 and 44 years of age [2].

Infection with the human papilloma virus (HPV) is the most significant risk factor for cervical cancer which can be transmitted during sexual intercourse. Based on Southern Asia studies performing HPV detection tests in cervical samples, about 7.9% of women in the general population are estimated to harbor cervical HPV infection at a given time, and 82.8% of invasive cervical cancers are attributed to HPVs 16 or 18 [1]. The other risk factors include early age at first intercourse, multiple sexual partners, poor sexual hygiene, repeated child birth smoking, immunodeficiency, having a history of cancer etc. [3].

In developed countries, programs are in place which enables women to get screened, making most pre-cancerous lesions identifiable at stages when they can easily be treated. Early treatment prevents up to 80% of cervical cancers in these countries [4].

However, in developing countries, only approximately 5% of eligible women undergo cytology-based screening in a 5-year period. Too few trained and skilled professionals for implementation, unavailability of healthcare resources to sustain the program, confinement of the services to teaching hospitals and private laboratories in urban areas and furthermore delay in reporting cytology results act as barriers that prevent cytology-based screening programs from being effective in developing countries [5].

Primary prevention measures, such as prophylactic vaccines against high risk HPV, are now available. However, the HPV vaccine is only effective if given before the commencement of sexual activity and concerns have been raised regarding the efficacy and usefulness of vaccination programs. Moreover, parental consent is required for children receiving the vaccine, which may challenge practitioners when educating parents. Over time, vaccination will decrease the prevalence of the disease among younger women but screening will still be needed [6].

The age-standardized annual incidence of cervical cancer in Nepal is 19.0 per 100,000, making Nepal a country with one of the highest cervical cancer rates in South Asia next to India (22 per 100,000) and Bangladesh (19.2 per 100,000), respectively. However, this estimate is most likely an underestimation of the true incidence of the disease due to a low number of cervical screening facilities and a lack of a national cancer registry. Though the Crude Incidence Rate of cervical cancer is 14.9 per 100,000, less than that of the World (15.1 per 100,000), the crude mortality rate is higher than that of the World (8.7 per 100,000 and 7.6 per 100,000, respectively) [2].

Over 80% of cervical cancers are diagnosed at an advanced clinical stage, which often have very poor prognosis [7]. Thus, regular cervical cancer screening is essential to decrease the incidence as well as mortality rate of cervical cancer.

Cervical cancer is the only preventable gynecological cancer. Yet Nepal has one of the highest incidence and mortality rates in South East Asian region. Early detection and prophylactic vaccines is the keystone for the prevention of cervical cancer screening. Pilot programs have been conducted for the prophylactic vaccines of cervical cancer but it does not cover all the oncogenic types of HPV and women who have already been exposed to HPV. Thus, screening programs still need to be focused.

It is worth highlighting that the knowledge of cervical cancer and availability of screening facilities are not adequate to promote the use of screening facility. There are several other factors that hinder women from attaining those services. Thus, a woman can have cervical cancer screening facility available in her locality, but this does not guarantee the uptake of facility for better health. Thus, this study aims to assess cervical cancer screening behavior and associated factors among women of residing in Nala Village Development Committee (VDC), Kavre.

## Methods

A quantitative descriptive study design was used to assess cervical cancer screening behavior and associated factors among women of Ugrachandi Nala VDC. Women of age 30–60 years were selected using systematic random sampling process. Women of age 30–60 years who were available at the time of the study and provided verbal consent for it were eligible for the study. A total of 180 respondents, who met eligible criteria were interviewed face to face. Women who were ever diagnosed with cervical cancer were excluded from the study.

Data were collected using a research tool based on Health Belief Model. The underlying concept of health belief model is that the health behavior is determined by personal beliefs or perceptions about disease and the strategies available to decrease its occurrence. The four main constructs of health belief model are as follows: perceived severity, perceived susceptibility, perceived benefits, and perceived barriers.

Data regarding education level of the participants were collected in six different categories namely, illiterate, can read and write, primary (class 1–5), secondary (class 6–10), higher secondary (+2), university, and above. But the assumptions of Chi-square test could not be met using these six categories; thus, the latter five categories were then merged into one category; literate, for drawing the inference.

Data were analyzed by using descriptive statistical method like percentage, frequency, mode. Chi-square tests were used to test the association between various categorical variables at 5% level of significance. The collected raw data were analyzed in SPSS 16 version for both descriptive and inferential statistics.

Written consent was obtained from the concerned authority. Verbal consent was taken from all respondents before data collection. Explanation about the purpose of study was given to the study population. The participants were allowed to withdraw from the study at any time without giving reason and without fear.

*Site:* In this VDC, the Nari Jagaran Sasthan associated with Family Planning Association of Nepal has been actively working to create awareness regarding several kinds of Cancer. Cervical cancer awareness program was also conducted 2 years back focusing to create awareness about cervical cancer and its screening measures. Cervical cancer screening campaigns have also been conducted in this area by the association as well as the local health personnel. Thus, it would be effective to measure the cervical cancer screening behavior among the women who already are aware regarding cervical cancer.

## Results

The sample involved 180 women between the ages of 30 and 60 years with the mean age being 42.64 ± 9.21 years. Out of 180 respondents, more than half (56.1%) were aged between 30 and 42 years. Majority (74.4%) of the respondents were literate with 27.8% who could read and write. Majority (38.3%) of the respondents were Newar, whereas minority (2.8%) of them was others. Majority of the respondents (99.4%) were Hindu by religion. More than half of the respondents (57.2%) were married at an age below 20 years with a mean age of 19.1 ± 3.67 years. Majority of the respondents (81.1%) had children ≤3, whereas 77.8% of women had no history of miscarriage. More than half (60%) of respondents had used family planning method. The most commonly used methods were Tubectomy (34.3%), Depo-Provera (28.7%), and Vasectomy (18.5%) (Table 1).

**Table 1:** Socio-demographic Information *n* = 180

Variables	Frequency (*f*)	Percentage (%)
Age (in years)		
≤42	101	56.1
>42	79	43.9
Education level		
Illiterate	46	25.6
Can read and write	50	27.8
Primary (class 1–5)	16	8.9
Secondary (class 6–10)	49	27.2
Higher secondary (+2)	17	9.4
University and above	2	1.1
Ethnicity		
Brahman	48	26.7
Chhetri	46	25.6
Newar	69	38.3
Dalit	12	6.7
Others	5	2.7
Religion		
Hindu	179	99.4
Christian	1	0.6
Age at marriage (in years)		
<20	103	57.2
≥20	77	42.8
Parity		
≤3	146	81.1
>3	34	18.9
History of miscarriage		
Yes	40	22.2
No	140	77.8
Use of family planning		
Yes	108	60
No	72	40
If yes, type of family planning mentioned*		
Norplant	6	5.6
Depo-Provera	31	28.7
Copper-T	9	8.3
Tubectomy	37	34.3
Vasectomy	20	18.5
Others	5	4.6

* *n* = 108

Table 2 illustrates that most of the respondents (63.3%) had ever had a gynecological examination. Majority of the respondents (94.4%) had heard about cervical cancer and (80.6%) had heard about cervical cancer screening. The most common (57.2%) source of information about cervical cancer screening behavior was health personnel.

**Table 2:** General information regarding cervical cancer screening *n* = 180

Variable	Frequency (*f*)	Percentage (%)
Ever had gynecological examination		
Yes	114	63.3
No	66	36.7
Heard about cervical cancer		
Yes	170	94.4
No	10	5.6
Heard about cervical cancer screening		
Yes	145	80.6
No	35	19.4
Source of information*		
Family and friends	38	26.2
Health personnel	83	57.2
Social media	24	16.6

* *n* = 145

Less than half (47.6%) of the respondents had ever been screened. Among those who were screened, the most common (69.6%) reason for screening was health personnel’s advice, whereas the least common (2.9%) reason was family’s advice. More than half of the respondents (56.5%) had been screened only once and less than half (49.3%) were screened within recent 3 years. Out of those who were last screened within recent 3 years, majority (91.3%) received the report of screening and among them the majority (96.8%) had sought consultation to show the report of screening. Minority of the respondents (47.5%) were suggested to repeat the test. Most of the respondents (44.9%) attended the screening facility from Private Hospitals and clinics, whereas least of the respondents (8.7%) from Government Hospitals (Table 3).

**Table 3:** Cervical cancer screening practice *n* = 145

Variable	Frequency (*f*)	Percentage (%)
Ever performed cervical cancer screening		
Yes	69	47.6
No	76	52.4
Reason for cervical cancer screening^a^		
Personal initiative	19	27.5
Health personnel’s advice	48	69.6
Family’s advice	2	2.9
Number of screenings done^a^		
Once	39	56.5
Twice	17	24.6
Thrice and more	13	18.8
Time of last screening^a^		
Before 3 years	35	50.7
Within 3 years	34	49.3
Received report^a^		
Yes	63	91.3
No	6	8.7
Report^b^		
Normal	60	95.2
Abnormal	3	4.8
Had consultation about report^b^		
Yes	61	96.8
No	2	3.2
Suggestion given^c^		
Repeat test	29	47.5
Colposcopy	2	3.3
Biopsy	2	3.3
No need to repeat	28	45.9
Service taken from		
Health post	21	30.4
Government hospital	6	8.7
Private clinics and hospital	31	44.9
Screening campaigns	19	27.5

^a^ *n* = 69

^b^ *n* = 63

^c^ *n* = 61

Out of total respondents, most of the respondents (81.7%) had no cervical cancer screening behavior, whereas only 18.3% of the respondents had cervical cancer screening behavior (Table 4).

**Table 4:** Cervical cancer screening behavior *n* = 180

Variable	Frequency (*f*)	Percentage (%)
Cervical cancer screening behavior		
Yes	33	18.3
No	147	81.7

In Tables 5 and 6, the items of health belief model scale for cervical cancer screening are shown according to their domains: perceived susceptibility, perceived severity, perceived benefits, and perceived barriers.

**Table 5:** Respondents’ responses to statements of beliefs regarding cervical cancer *n* = 180

S. no	Variables	SA	A	N	D	SD
		*f* (%)	*f* (%)	*f* (%)	*f* (%)	*f* (%)
1	I feel I will get cervical cancer in the future.	7 (3.9)	27 (15)	46 (25.6)	26 (14.4)	74 (41.1)
2	I am more likely than the average woman to get cervical cancer	6 (3.3)	7 (3.9)	25 (13.9)	43 (23.9)	99 (55.0)
3	Cervical cancer only happens to women over 50 years	11 (6.1)	22 (12.2)	48 (26.7)	78 (43.3)	21 (11.7)
4	Young women are at risk for cervical cancer.	57 (31.7)	69 (38.3)	37 (20.6)	11 (6.1)	6 (3.3)
5	Cervical cancer is not as serious as other types of cancer.	31 (17.2)	20 (11.1)	36 (20.0)	16 (8.9)	77 (42.8)
6	Cervical cancer is easily cured.	26 (14.4)	45 (25.0)	40 (22.2)	24 (13.3)	45 (25.0)
7	There are effective treatments for cervical cancer.	45 (25.0)	85 (47.2)	40 (22.2)	1 (0.6)	9 (5.0)
8	I am afraid to think about cervical cancer.	91 (50.6)	41 (22.8)	8 (4.4)	22 (12.2)	18 (10.0)
9	Problems I would experience with cervical cancer would last a long time	84 (46.7)	32 (17.8)	45 (25.0)	14 (7.8)	5 (2.8)
10	Cervical cancer would threaten a relationship with my husband or partner	100 (55.6)	30 (16.7)	38 (21.1)	5 (2.8)	7 (3.9)
11	If I had cervical cancer my whole life would change	77 (42.8)	59 (32.8)	25 (13.9)	13 (7.2)	6 (3.3)
12	If I developed cervical cancer, I would not live longer than 5 years	59 (32.8)	11 (6.1)	50 (27.8)	33 (18.3)	27 (15)

*SD* strongly disagree, *D* disagree, *N* neutral, *A* agree, *SA* strongly agree

**Table 6:** Respondents’ responses to statements of beliefs regarding cervical cancer screening *n* = 180

S. no.	Variables	SA	A	N	D	SD
		*f* (%)	*f* (%)	*f* (%)	*f* (%)	*f* (%)
1	When I complete cervical cancer screening I don’t worry as much about cervical cancer	84 (46.7)	31 (17.2)	24 (13.3)	23 (12.8)	18 (10.0)
2	Completing cervical cancer screening will allow me to find out if there are early signs of cervical cancer	123 (68.3)	24 (13.3)	25 (13.9)	4 (2.2)	4 (2.2)
3	If I complete cervical cancer screening, I will decrease my chances of dying from cervical cancer	118 (65.6)	25 (13.9)	30 (16.7)	4 (2.2)	3 (1.7)
4	If I complete cervical cancer screening, I will decrease my chance of pain and surgery related to cervical Cancer	82 (45.6)	51 (28.3)	39 (21.7)	8 (4.4)	0 (0.0)
5	Undergoing cervical cancer screening will make me worry about cervical cancer	63 (35.0)	18 (10.0)	33 (18.3)	24 (13.3)	42 (23.3)
6	Cervical cancer screening will be embarrassing to me	96 (53.3)	21 (11.7)	3 (1.7)	29 (16.1)	31 (17.2)
7	I don’t know where I could go if I wanted cervical cancer screening.	86 (47.8)	26 (14.4)	7 (3.9)	17 (9.4)	44 (24.4)
8	Cervical cancer screening is too expensive.	41 (22.8)	14 (7.8)	74 (41.1)	21 (11.7)	30 (16.7)
9	Undergoing cervical cancer screening will take too much time	33 (18.3)	9 (5.0)	69 (38.3)	25 (13.9)	44 (24.4)
10	Cervical Cancer Screening procedure will be painful	63 (35.0)	32 (17.8)	49 (27.2)	17 (9.4)	19 (10.6)
11	Cervical Cancer Screening will interfere with my family obligations	20 (11.1)	6 (3.3)	16 (8.9)	78 (43.3)	60 (33.3)
12	I prefer females perform cervical cancer screening because it is uncomfortable to me if a man does it.	145 (80.6)	13 (7.2)	5 (2.8)	6 (3.3)	11 (6.1)

*SD* strongly disagree, *D* disagree, *N* neutral, *A* agree, *SA* strongly agree

More than half of the participants (55.0%) strongly disagreed on the item “I am more likely than the average woman to get cervical cancer” in the domain perceived susceptibility with the modal value of 99, whereas more than half of the participants (55.6%) strongly agreed on the item “Cervical cancer would threaten a relationship with my husband or partner” in the domain perceived severity with modal value of 100.

The item “Completing cervical cancer screening will allow me to find out if there are early signs of cervical cancer” in the domain perceived benefits was strongly agreed to by more than half of the participants (68.3%) with the modal value of 123, while most of the participants (80.6%) strongly agreed on the item “I prefer females perform cervical cancer screening because it is uncomfortable to me if a man does it” in the domain perceived barriers with modal value of 145.

Table 7 illustrates that only 11.7% of respondents had high perceived susceptibility, 44.4% had high perceived severity, whereas more than half (74.4%) had high perceived benefits and less than half (32.2%) had high perceived barriers.

**Table 7:** Perception regarding cervical cancer and cervical cancer screening *n* = 180

Variables	High *F* (%)	Low *F* (%)
Perceived susceptibility	21 (11.7)	159 (88.3)
Perceived severity	80 (44.4)	100 (55.6)
Perceived benefits	134 (74.4)	46 (25.6)
Perceived barriers	58 (32.2)	122 (67.8)

There is no association between cervical cancer screening behavior and age of the respondents and cervical cancer screening behavior and parity of the respondents. Therefore, it was concluded that cervical cancer screening behavior of the respondents was independent of age of respondents and parity of respondents. Table 8 also shows that there is significant association between cervical cancer screening behavior and education level of the respondents. Therefore, null hypothesis (there is no significant association between educational level and cervical cancer screening behavior among women of Ugrachandi Nala VDC, Kavre) is rejected and research hypothesis is accepted (Table 8).

**Table 8:** Association between age, education, and parity of respondents and cervical cancer screening behavior *n* = 180

Socio-demographic variables	Cervical cancer screening behavior		Total	*p*
	Yes *F* (%)	No *F* (%)		
Age (in years)				
≤42	20 (19.8)	81 (80.2)	101 (100.0)	0.698
>42	13 (16.5)	66 (83.5)	79 (100.0)	
Education level				
Literate	30 (22.4)	104 (77.6)	134 (100.0)	0.015*
Illiterate	3 (6.5)	43 (93.5)	46 (100.0)	
Parity				
≤3	30 (20.5)	116 (79.5)	146 (100.0)	0.142
>3	3 (8.8)	31 (91.3)	34 (100.0)	

* Significant

Table 9 illustrates that there is no association between cervical cancer screening behavior and perceived severity of cervical cancer. Therefore, it was concluded that cervical cancer screening behavior of the respondents was independent of perceived severity of cervical cancer.

**Table 9:** Association between perceived severity of cervical cancer and cervical cancer screening behavior *n* = 180

Perceived severity	Cervical cancer screening behavior		Total	*p*
	Yes *F* (%)	No *F* (%)		
High	18 (22.5)	62 (77.5)	80 (100.0)	0.245
Low	15 (15.0)	85 (85.0)	100 (100.0)	
Total	33 (18.3)	147 (81.7)	180 (100.0)	

Table 10 shows that there is no association between cervical cancer screening behavior and perceived benefits of cervical cancer screening behavior. Therefore, it was concluded that cervical cancer screening behavior of the respondents was independent of perceived benefits of cervical cancer.

**Table 10:** Association between perceived benefits of cervical cancer screening and cervical cancer screening behavior *n* = 180

Perceived benefits	Cervical cancer screening behavior		Total	*p*
	Yes *F* (%)	No *F* (%)		
High	29 (21.6)	105 (78.4)	139 (100.0)	0.075
Low	4 (8.7)	42 (91.3)	46 (100.0)	
Total	33 (18.3)	147 (81.7)	180 (100.0)	

Table 11 illustrates that there is no association between cervical cancer screening behavior and perceived barriers of cervical cancer screening behavior. Therefore, it was concluded that cervical cancer screening behavior of the respondents was independent of perceived barriers of cervical cancer.

**Table 11:** Association between perceived barriers of cervical cancer screening and cervical cancer screening behavior *n* = 180

Perceived barriers	Cervical cancer screening behavior		Total	*p*
	Yes *F* (%)	No *F* (%)		
High	8 (13.8)	50 (86.2)	58 (100.0)	0.310
Low	25 (20.5)	97 (79.5)	122 (100.0)	
Total	33 (18.3)	147 (81.7)	180 (100.0)	

## Discussion

In our study, majority of the respondents had heard about cervical cancer. Similar result has been shown in a study conducted by Shrestha et al. [8], Nepal and other studies conducted in North Eastern India and North Central Nigeria [8–10]. The fact that the study was conducted in an area where awareness programs have been conducted on cervical cancer might be the reason for a large population of women having heard about cervical cancer.

This study found out that majority of the women had also heard about cervical cancer screening. More than half of the women (57.2%) who had heard about cervical cancer screening stated that they got the information from health personnel followed by family and friends (26.2%) and social media (16.6%). This is similar to that reported by another study by Singh in India where health personnel followed by friends was the source of information [11]. Another study by Shrestha reported family and friends to be the main source of information [8].

In this study, similar finding has been found as that of the study conducted by Ibekwe et al. [12]. In both of the studies, only minorities of women have ever been screened for cervical cancer. In the study conducted by Ibekwe et al. [12], the cervical cancer screening status was found to be 39% which is similar to present study in which 47.6% of women have ever been screened for cervical cancer. This is still lower than the national target coverage which is 50% in women of age 30–60 years [13]. Similarly, in a study conducted by Sawadogo et al., minority of women were found to be screened for cervical cancer [14]. In contrast to the present study, majority of the Vietnamese Americans were found to have screened for cervical cancer in a study conducted by Grace et al. [15].

This study showed that the reason why more than half of the women (69.6%) that had ever been screened for cervical cancer was health personnel’s advice followed by personal initiative (27.5%) and family’s advice (2.9%). A study conducted by Singh asserted prevention of cervical cancer (54.5%) as the main reason for adequate practice followed by health personnel’s advice (15.2%) and family’s advice (12.2%) [11].

The results of this study showed that minority of the women had cervical cancer screening behavior, i.e., 18.3%. This is in keeping with the study conducted by John in Ruvuma in which only 14% of the respondents had good cervical cancer screening practice [16]. On the bright side, the finding of this study is higher than 2.0% that was reported in national cervical cancer screening coverage among rural women (2014) [2]. This might be explained by the fact that the local leaders in our study area have been involved in increasing the awareness of cervical cancer and utilization of cervical cancer screening services, and therefore increasing ease of access to the cervical cancer screening service.

This study demonstrated a significant relationship between cervical cancer screening behavior and education level of respondents (*p* = 0.015) which is in accordance to the study conducted by Morema et al. in Kenya with the *p* < 0.0001 [17]. Similarly, a study conducted by Tracy in USA showed that routine screeners were more likely to have graduated college (p = 0.01) [18]. A study conducted by Hyacinth in North Central Nigeria showed statistically significant relationship of Educational Level and utilization of Pap smear test (*p* = 0.01) [10]. But the study is in contrast to the study conducted by Shrestha et al. (*p* = 0.081) [8]. In this study, cervical cancer screening behavior has been found to be high among literate women. This fact may be due to literate women being more aware about cervical cancer and its prevention and conscious regarding their well-being.

No significant association has been found between age of women and cervical cancer screening behavior (*p* = 0.698). This is in contrast to studies conducted by Shrestha et al. in Nepal (*p* = 0.013), [8] Hyacinth et al. in North Central Nigeria (*p* < 0.01) [10], Esin et al. in Turkey (*p* = 0.000) [19] and Morema in Kenya (*p* < 0.0001) [17]. Such facts may be due to the screening campaign being successful enough to involve women of every age group. It might be because older women tend to perform screening more on health personnel’s recommendation and younger women on their own initiative to remain healthy. Thus, the screening behavior is independent of age.

From the present study, no significant association has been found between parity of women and cervical cancer screening behavior (*p* = 0.142). This is in accordance to a study conducted by Hyacinth et al. in North Central Nigeria (*p* = 0.28) [10] and study conducted by Shrestha et al. Nepal (*p* = 0.153) [8]. But it can be considered that the higher parity the women has, the higher probability of contact with the health personnel, thus increasing the recommendation of the screening.

In this study, no significant association was found between cervical cancer screening behavior and perceived severity (*p* = 0.245) which is in accordance to studies conducted by Ibekwe et al. in Botswana (*p* = 0.2988) [20] and Esin et al. in Turkey [19]. In contrast to present study, a study conducted by Morema [17] in Kenya showed that those women who perceived cervical cancer as a serious disease were significantly more likely to report having had a cervical screen (*p* < 0.0001) [17]. Concerning perceived severity, most women believed that cervical cancer would threaten their relationship with their husband or partner (55.6%) and that they were afraid to think about cervical cancer (50.6%).

In this study, no significant association was found between cervical cancer screening behavior and perceived benefits (*p* = 0.075). Similar studies conducted by Ibekwe et al. in Botswana (*p* = 0.2988) [21] and Esin et al. [19] in Turkey have shown no significant association between cervical cancer screening and perceived benefits. Concerning perceived benefits, majority of the women (68.3%) believed that completing cervical cancer screening will allow them to find out if there are early signs of cervical cancer.

Since the women may have attended similar awareness programs in the past regarding cervical cancer and cervical cancer screening behavior, it may have influenced perceived severity of cervical cancer and perceived benefits of cervical cancer screening in the same way in both groups of women who have and who do not have cervical cancer screening behavior.

Similarly, this study showed no significant association between cervical cancer screening behavior and perceived barriers (*p* = 0.310). Similar study conducted by Ibekwe et al. [21] in Botswana (*p* = 0.696) has shown no significant association between cervical cancer screening and perceived barriers [12]. It can be considered that higher the perceived barriers of cervical cancer screening, lower will be the screening rates. However, in our context, health personnel’s advices regarding examination have a great role. Even though when women may have had high perceived barriers, health personnel’s advice may have led to cervical cancer screening behavior. Concerning perceived barriers, majority of the women (80.6%) believed that they prefer that females perform cervical cancer screening because it is uncomfortable to them if a man does it.

## Conclusion

This study shows that cervical cancer screening behavior among the respondents was satisfactory. The major reason for cervical cancer screening was health personnel’s advice. Women who were literate were found to have significantly higher cervical cancer screening behavior as compared to their reference. Minority of women perceived themselves at risk of cervical cancer. Majority of women felt uncomfortable to be screened by male health personnel. Still only minority of the respondents were suggested to repeat cervical cancer screening by health personnel in future. No significant association was found between cervical cancer screening behavior and age, parity, perceived severity, perceived benefits, and perceived barriers.

### Limitation

As the respondents had to remember when they had performed cervical cancer screening, there might have been recall bias. In this study, association between perceived susceptibility and cervical cancer screening behavior could not be assessed because of error caused by small sample size for Chi-square test.

### Implication

This study to assess cervical cancer screening behavior is conducted for the first time in this area. Hence, it could provide help for further research to those conducting future research. The study might be useful for planning new intervention or education for cervical cancer screening.

### Recommendation

This study offers recommendations for health professionals, nurses, and community health workers to focus preventive health measures for cervical cancer prevention.

Awareness campaigns targeting illiterate groups can be conducted in community so that they become motivated towards cervical cancer screening.

Since the most common source of information was health personnel and the most common reason for screening was their advice. For this health professionals can conduct health education programs to the women eligible for screening attending gynecological clinic regarding prevention of cervical cancer and importance of cervical cancer screening. So that it can boost up their preventive behaviors and cervical cancer screening behavior.

The health personnel could also provide women with choice from whom they want to be screened. In such situation, nurses being female in our context could come forward so that they do not miss the opportunity to be screened.
